# Characteristics of patients with atrial high rate episodes detected by implanted defibrillator and resynchronization devices

**DOI:** 10.1093/europace/euab186

**Published:** 2021-08-24

**Authors:** Kazuo Miyazawa, Daniele Pastori, David T Martin, Wassim K Choucair, Jonathan L Halperin, Gregory Y H Lip, the IMPACT Study Investigators

**Affiliations:** 1 Department of Cardiovascular Medicine, Chiba University Graduate School of Medicine, 1-8-1 Inohana Chuo-ku, Chiba 260-8677, Japan; 2 Department of Clinical Internal, Anesthesiological and Cardiovascular Sciences, Sapienza University of Rome, Piazzale Aldo Moro, Rome 5 00185, Italy; 3 Liverpool Centre for Cardiovascular Science, University of Liverpool and Liverpool Heart & Chest Hospital, Thomas Drive, Liverpool L14 3PE, UK; 4 Department of Medicine, Brigham and Women’s Hospital, Boston, 41 Mall Road Burlington, MA 01805, USA; 5 Heart and Rhythm Institute of South Texas, 8122 Datapoint Dr, San Antonio, TX 78229, USA; 6 Department of Cardiovascular Diseases, Icahn School of Medicine at Mount Sinai, Mount Sinai Hospital, Mount Sinai Heart, 1 Gustave L. Levy Pl, New York, NY 10029, USA; 7 Aalborg Thrombosis Research Unit, Department of Clinical Medicine, Aalborg University, Fredrik Bajers Vej 7K, Aalborg Øst 9220, Denmark

**Keywords:** Atrial fibrillation, Atrial high rate episode, Stroke, Thromboembolism, Clinical profile, Cardiac implantable electronic device

## Abstract

**Aims:**

Atrial high rate episodes (AHREs) are associated with increased risks of thromboembolism and cardiovascular mortality. However, the clinical characteristics of patients developing AHRE of various durations are not well studied.

**Methods and results:**

This was an ancillary analysis of the multicentre, randomized IMPACT trial. In the present analysis, we classified patients according to the duration of AHRE ≤6 min, >6 min to ≤6 h, >6 to ≤24 h and >24 h, and investigated the association between clinical factors and the development of each duration of AHRE. Of 2718 patients included in the trial, 945 (34.8%) developed AHRE. The incidence rates of each AHRE duration category were 5.4/100, 12.0/100, 6.8/100, and 3.3/100 patient-years, respectively. The incidence rates of AHRE >6 h were significantly higher in patients at high risk of thromboembolism (CHADS_2_ score ≥3) compared to those at low risk (CHADS_2_ score 1 or 2). Using Cox regression analysis, age ≥65 years and history of atrial fibrillation (AF) and/or atrial flutter (AFL) were risk factors for AHRE >6 min. In addition, hypertension was associated with AHRE >24 h (hazard ratio 2.13, 95% confidence interval 1.24–3.65, *P *=* *0.006).

**Conclusion:**

Atrial high rate episode >6 min to ≤6 h were most prevalent among all AHRE duration categories. Longer AHREs were more common in patients at risk of thromboembolism. Age and history of AF/AFL were risk factors for AHRE >6 min. Furthermore, hypertension showed a strong impact on the development of AHRE >24 h rather than age.

What’s new?

- Atrial high rate episode (AHRE) lasting >6 min to ≤6 h most frequently occurred among duration-categorized AHREs in patients at risk of life-threatening arrhythmias.
- Atrial high rate episodes lasting >6 h more frequently occurred in patients at high risk of thromboembolism compared to those at low risk.
- Age and history of atrial fibrillation or flutter were risk factors associated with the development of AHREs lasting >6 min.
- Hypertension was a risk factor for the development of long AHRE (>24 h), with a greater impact than age.

## Introduction

Atrial fibrillation (AF) is associated with increased risks of stroke, myocardial infarction, heart failure, and mortality.^1^ A substantial proportion of AF is asymptomatic and often eludes detection by conventional diagnostic methods such as physical examination, 12-lead electrocardiogram (ECG), and 24-h Holter ECG.^2^ In approximately one-quarter of patients with stroke, no overt aetiology is identified, and these events may be related to atrial high rate episodes (AHREs), which generally represent either subclinical AF or atrial flutter (AFL).^3^

Cardiac implantable electronic devices (CIEDs) can automatically record all spontaneous episodes of atrial and ventricular arrhythmias regardless of symptoms. Previous studies demonstrated that AHRE is associated with increased risks of developing clinically manifest AF,^4^ thromboembolism,^5^^,^^7^ cardiovascular events,^8^ and mortality.^9^ Atrial high rate episode lasting more than 5 or 6 min is considered clinically relevant, and patients presenting with AHRE should be assessed for other stroke risk factors.^5,^^9^ Thus, current clinical practice guidelines address diagnosis and management of patients with AHRE.^10^ Although inflammation at the time of CIED implantation is associated with subsequently detected AHRE,^11^ the clinical profiles of patients with AHRE of various durations have not been defined.

The IMPACT trial included 2718 patients with implantable cardioverter-defibrillators (ICDs) or cardiac resynchronization therapy (CRT) devices, and examined the strategy of basing anticoagulation therapy on the burden of AHRE and each patient’s intrinsic risk of thrombeoembolism.^13^ In a sub-study of the trial, we sought to identify clinical characteristics of patients with various burdens of AHRE and to assess the risk factors associated with the development of AHRE of various durations in patients with ICD or CRT-D devices.

## Methods

### Study population

The study design of IMPACT (ClinicalTrials.gov identifier NCT00559988) has been previously described.^12^ In brief, this interventional, single-blinded, randomized, multicentre trial enrolled 2718 patients with dual-chamber ICD or CRT-D devices (Biotronik, Inc.) at 104 centres in North America, Europe, and Australia. Eligible patients had at least one additional stroke risk factor (CHADS_2_ score ≥1) and were deemed able to tolerate anticoagulation. Patients with permanent AF or contraindications to anticoagulation were excluded. Patients were randomized to a strategy of starting and stopping anticoagulation based on remote rhythm monitoring vs. usual office-based follow-up with anticoagulation based on standard clinical criteria. The protocol was approved by institutional review boards governing human research.

### Definition of atrial high rate episode

Atrial high rate episode was defined as atrial tachyarrhythmias with ≥36 of 48 atrial beats and cycle lengths ≤300 ms (atrial rates ≥200 b.p.m.). For this analysis, we subdivided AHRE into five categories according to duration as no AHRE, episodes ≤6 min, >6 min but ≤6 h, >6 h but ≤24 h, and those lasting >24 h. Classification was based on the longest AHRE during follow-up (median 701 days and cumulative 5430 patient-years).

### Statistical analysis

Continuous variables were expressed as mean ± standard deviation, and categorical variables as numbers and percentages. We compared categorical variables using the *χ*^2^ test and continuous variables using the independent samples *t*-test for normally distributed data or Mann–Whitney *U* test for non-normal distribution. Significance was accepted at the 95% confidence interval (CI, two-sided *P* ≤ 0.05). Since we performed multiple (four pairwise) comparisons of baseline characteristics, the Bonferroni correction was applied to adjust the threshold for significance (0.05/4 = 0.0125).

To identify independent risk factors associated with AHRE, we performed Cox proportional hazards regression analysis. The multivariable models were adjusted for underlying heart disease, medications, and components of the CHA_2_DS_2_-VASc score as covariates. The cumulative incidence of AHRE of various durations was displayed using the Kaplan–Meier method. Receiver operating characteristic (ROC) analysis was performed to evaluate the discrimination of the risk scores to predict the development of AHRE based on the area under the ROC curves (AUC). To compare the predictive models, we calculated the difference between the AUCs by the method of DeLong *et al*.^13^

Statistical analyses were performed using SAS version 9.2 (SAS Institute Inc., Cary, NC, USA), SPSS version 21 (IBM Corp., Armonk, NY, USA), and StatXact version 10 (Cytel, Cambridge, MA, USA).

## Results

### Baseline patient characteristics

Baseline characteristics of enrolled patients are shown in *Table 1*. Mean age was 64.4 years; 26.3% were women. Of all patients, 64% had ICD and 36% CRT-D devices. During a mean follow-up of 2.0 ± 1.2 years, 945 patients (34.8%) developed AHRE; 292 (10.7%) had AHRE ≤6 min, 284 (10.4%) >6 min to ≤6 h, 187 (6.9%) >6 to ≤24 h, and 182 (6.7%) >24 h.

**Table 1:** Baseline characteristics of patients

	No AHRE group (*n* = 1773)	AHREs groups							
		≤6 min (*n* = 292)	*P*-value	>6 min/≤6 h (*n* = 284)	*P*-value^a^	>6 h/≤24 h (*n* = 187)	*P*-value^a^	>24 h (*n* = 182)	*P*-value^a^
Age (years)	63.9 ± 11.2	62.4 ± 11.1	0.050	66.8 ± 10.6	<0.001	66.8 ± 10.6	<0.001	66.5 ± 10.6	0.001
Women	490 (27.6)	86 (29.5)	0.522	61 (21.5)	0.030	38 (20.3)	0.032	40 (22.0)	0.102
Hypertension	1480 (83.5)	243 (83.2)	0.914	227 (79.9)	0.140	160 (85.6)	0.463	167 (91.8)	0.003
Diabetes mellitus	733 (41.3)	111 (38.0)	0.284	107 (37.7)	0.243	74 (39.6)	0.243	84 (46.2)	0.210
Heart failure	1607 (90.6)	252 (86.3)	0.022	257 (90.5)	0.938	164 (87.7)	0.938	165 (90.7)	0.992
History of stroke/TIA	159 (9.0)	34 (11.6)	0.146	23 (8.1)	0.632	14 (7.5)	0.632	13 (7.1)	0.408
Vascular disease	1035 (58.4)	157 (53.7)	0.140	165 (58.1)	0.930	115 (61.5)	0.930	112 (61.5)	0.409
Non-ischaemic CM	617 (34.8)	119 (40.8)	0.049	106 (37.3)	0.408	65 (34.8)	0.991	55 (30.2)	0.215
Valvular disease	899 (50.7)	160 (54.8)	0.198	158 (55.6)	0.125	101 (54.0)	0.394	106 (58.2)	0.054
History of AF/AFL	138 (7.8)	29 (9.9)	0.212	52 (18.3)	<0.001	48 (25.7)	<0.001	63 (34.6)	<0.001
CHADS_2_ score	2.5 ± 1.0	2.5 ± 1.1	0.341	2.5 ± 1.1	0.353	2.5 ± 1.1	0.917	2.6 ± 1.0	0.074
CHA_2_DS_2_-VASc score	3.9 ± 1.5	3.7 ± 1.4	0.070	3.9 ± 1.6	0.934	3.9 ± 1.5	0.492	4.1 ± 1.4	0.055
ICD	1095 (61.8)	211 (72.3)	0.001	186 (65.5)	0.228	128 (68.5)	0.073	112 (61.5)	0.953
Primary prevention of SCD	1566 (88.3)	247 (84.6)	0.071	250 (88.0)	0.885	164 (87.7)	0.801	164 (90.1)	0.474
Beta-blockers	1628 (91.8)	267 (91.4)	0.825	254 (89.4)	0.181	165 (88.2)	0.095	165 (90.7)	0.588
ACE-I/ARB	1467 (82.7)	253 (86.6)	0.098	239 (84.2)	0.557	162 (86.6)	0.177	155 (85.2)	0.408
Digoxin	230 (13.0)	39 (13.4)	0.857	47 (16.6)	0.101	29 (15.5)	0.330	34 (18.7)	0.032
AAD	188 (10.6)	25 (8.6)	0.288	30 (10.6)	0.984	19 (10.2)	0.175	26 (14.3)	0.130
Statin	1310 (73.9)	200 (68.5)	0.054	205 (72.2)	0.545	144 (77.0)	0.355	140 (76.9)	0.373
Follow-up periods (days)^b^	NA	827.7 ± 426.9	Ref	830.4 ± 441.0	0.926	907.1 ± 443.7	0.053	936.9 ± 436.2	0.008

AAD, antiarrhythmic drugs; ACE-I, angiotensin-converting enzyme inhibitor; AF, atrial fibrillation; AFL, atrial flutter; AHRE, atrial high rate episode; ARB, angiotensin II receptor blocker; CM, cardiomyopathy; ICD, implantable cardioverter-defibrillator; NA, not applicable; SCD, sudden cardiac death; TIA, transient ischaemic attack.

a Versus no AHRE group.

b Versus AHRE ≤6 min.

Patients developing AHRE for ≤6 min were slightly younger, with less heart failure but a higher prevalence of non-ischaemic cardiomyopathy and higher proportion of ICD devices (*Table 1*). Patients with AHRE lasting >6 min to ≤6 h and >6 to ≤24 h were older and less frequently women than patients without AHRE. Conversely, a history of AF or AFL was more frequent in these groups. Patients with AHRE >24 h were older than those without AHRE but a similar proportion were women. In this group, there was a higher prevalence of AF or AFL at baseline and more frequent use of digoxin (*Table 1*).

**Figure 1:**
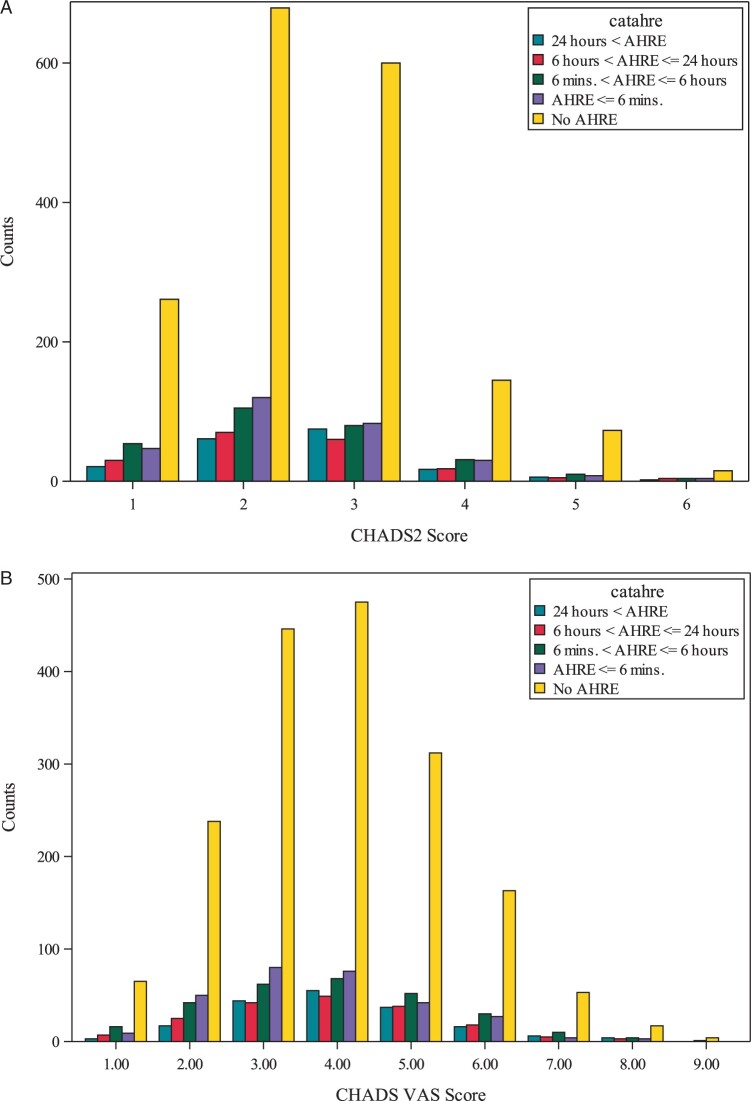
The distribution of patients with CIED according to the risk scores [(*A*) CHADS_2_ score and (*B*) CHA_2_DS_2_-VASc score]. AHRE, atrial high rate episode.

Of those who developed AHRE, 64.6% had CHADS_2_ scores of 2 and 26.2% had CHA_2_DS_2_-VASc scores of 4 (*Figure 1*).

### Incidence rates of atrial high rate episode

**Figure 2:**
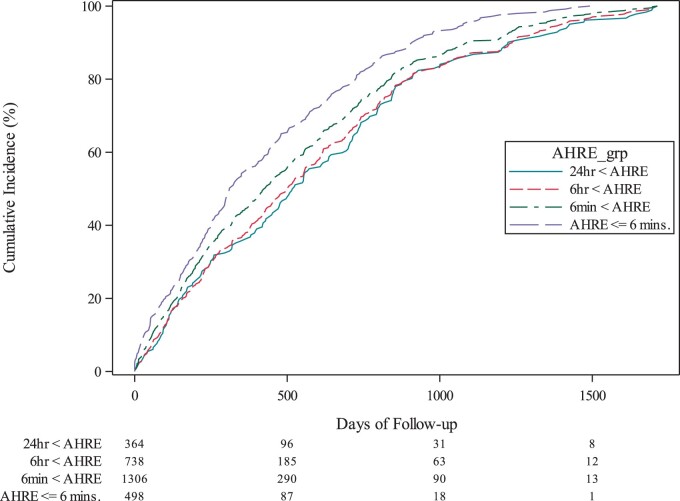
The cumulative incidence of different burden of AHRE using the Kaplan–Meier method. AHRE, atrial high rate episode.

The Kaplan–Meier estimates of the cumulative incidence of each AHRE duration are depicted in *Figure 2*. The incidence rates of each AHRE category during follow-up were 5.4/100, 12.0/100, 6.8/100, and 3.3/100 patient-years, respectively (*Table 2*). Time frame over which AHREs were documented was described in Supplementary material online, *Table* *S1*. To assess the relationship between incidence rate and thromboembolic risk, we divided patients into two groups according to CHADS_2_ score—those at low risk (CHADS_2_ score 1 or 2) and those at high risk (CHADS_2_ score ≥3)—and compared the incidence rates of each AHRE category in these two groups (*Table 2*). There were no significant differences between low-risk and high-risk patients in the incidence rates of AHRE ≤6 min and >6 min to ≤6 h [risk ratio (RR) 0.906, 95% CI 0.728–1.128, RR 1.108, 95% CI 0.969–1.266], while the incidence rates of AHRE >6 to ≤24 h, and those lasting >24 h were higher in high-risk patients compared to those with low-risk CHADS_2_ scores (RR 1.244, 95% CI 1.029–1.504 and RR 1.476, 95% CI 1.113–1.957, respectively).

**Table 2:** Incidence rate of each burden of AHREs

	Overall		Low-risk group (CHADS_2_ = 1 or 2)		High-risk group (CHADS_2_ ≥ 3)		RR (95% CI)^a^
	Number of patients	Incidence rate (100 patient-years)	Number of patients	Incidence rate (100 patient-years)	Number of patients	Incidence rate (100 patient-years)	
AHRE ≤ 6 min	292	5.364	167	5.60	125	5.076	0.906 (0.73–1.13)
6 min < AHRE ≤ 6 h	653	11.995	341	11.439	312	12.669	1.11 (0.97–1.27)
6 h < AHRE ≤ 24 h	369	6.778	182	6.105	187	7.593	1.24 (1.03–1.50)
AHRE > 24 h	182	3.343	82	2.751	100	4.061	1.48 (1.11–1.96)
	Overall		Low-risk group (CHA_2_DS_2_-VASc = 1–3)		High-risk group (CHA_2_DS_2_-VASc ≥ 4)		RR (95% CI)^a^
	Number of patients	Incidence rate (100 patient-years)	Number of patients	Incidence rate (100 patient-years)	Number of patients	Incidence rate (100 patient-years)	
AHRE ≤ 6 min	292	5.364	139	5.888	153	4.963	0.84 (0.68–1.05)
6 min < AHRE ≤ 6 h	653	11.995	258	10.929	395	12.812	1.17 (1.02–1.34)
6 h < AHRE ≤ 24 h	369	6.778	138	5.846	231	7.493	1.28 (1.05–1.56)
AHRE > 24 h	182	3.343	64	2.711	118	3.828	1.41 (1.05–1.90)

AHRE, atrial high rate episode; CI, confidence interval; RR, risk ratio.

a Low-risk group as a reference.

Similarly, we assessed this relationship using CHA_2_DS_2_-VASc score and observed the consistent results with the results using CHADS_2_ score, showing significantly high incidence rates of AHRE >6 h in high-risk patients (CHA_2_DS_2_-VASc score ≥4) compared to those at low risk (CHA_2_DS_2_-VASc score 1–3) (RR 1.282, 95% CI 1.053–1.560 for AHRE >6 to ≤24 h, RR 1.412, 95% CI 1.052–1.896 for AHRE >24 h). Furthermore, AHRE >6 min to ≤6 h also occurred more frequently in high-risk patients compared to those at low risk (RR 1.172, 95% CI 1.022–1.345).

In order to assess the relationship between the development of AHRE over time, we compared the follow-up duration among four AHRE categories. *Table 1* demonstrates that the follow-up duration in patients with AHRE >24 h was significantly longer than those with AHRE <6 min (*P *=* *0.008), while there was no significant difference in the follow-up duration among other AHRE duration subgroups.

### Risk factors for the development of atrial high rate episode

Cox regression analysis found heart failure inversely associated with the risk of AHRE ≤6 min [hazard ratio (HR) 0.58, 95% CI 0.40–0.85, *P *=* *0.005]. Conversely, age ≥65 years and a history of AF or AFL were associated with AHRE of durations longer than 6 min (*Figure 3A–D*). Female gender was inversely associated with AHRE >6 min to ≤6 h (HR 0.72, 95% CI 0.54–0.96, *P *=* *0.027) and >6 to ≤24 h (HR 0.70, 95% CI 0.49–1.02, *P *=* *0.061). Hypertension was associated with AHRE >24 h (HR 2.13, 95% CI 1.24–3.65, *P *=* *0.006).

**Figure 3:**
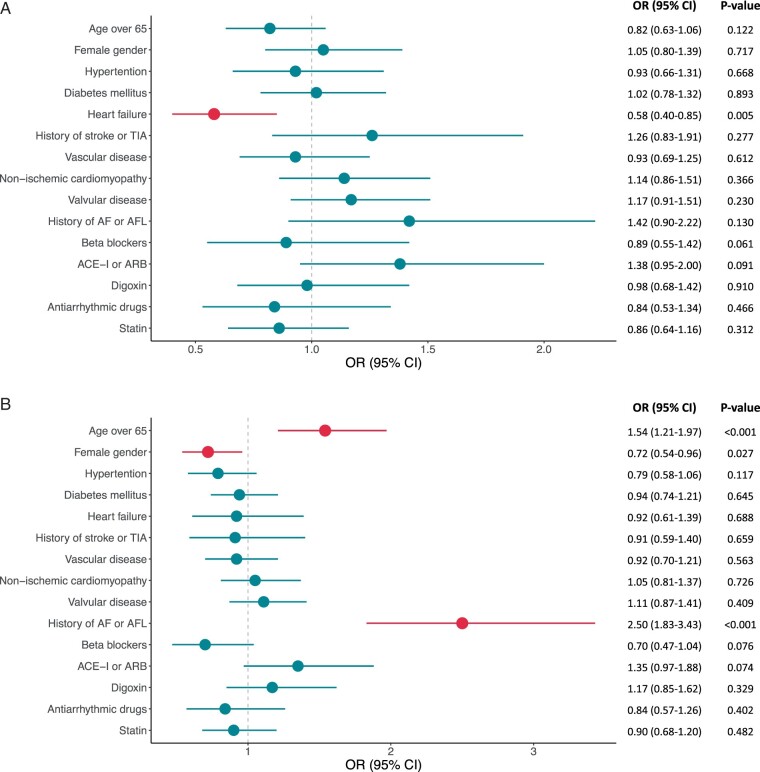
Clinical factors associated with the development of each burden of AHRE [(*A*) AHRE <6 min, (*B*) 6 min < AHRE ≤ 6 h, (*C*) 6 h < AHRE ≤ 24 h, and (*D*) AHRE > 24 h]. ACE-I, angiotensin-converting enzyme inhibitor; AF, atrial fibrillation; AFL, atrial flutter; ARB, angiotensin II receptor blocker; CI, confidence interval; CM, cardiomyopathy; HR, hazard ratio; TIA, transient ischaemic attack.

**Figure 3d:**
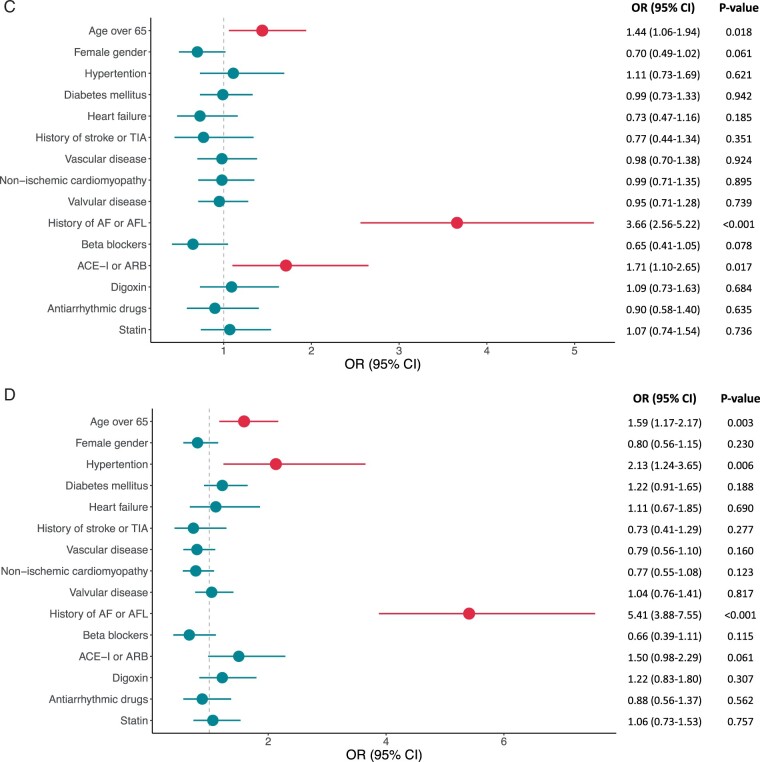


Additionally, we performed a subgroup analysis in patients without history of AF or AFL. In general, the consistent results of associations between AHRE and clinical factors were observed with the analysis for the entire population (Supplementary material online, *Table* *S2*). Several associations of long AHRE (>24 h) with age ≥65 years and hypertension did not reach the statistically significant threshold in this analysis. This was in part due to a relative decrease in the number of patients with AHRE, although we found a trend for age ≥65 years and hypertension being associated with long AHRE (HR 1.44, 95% CI 0.99–2.09, *P* = 0.054 for age ≥65 years, HR 1.87, 95% CI 0.97–3.6, *P* = 0.063 for hypertension).

### Association of CHADS_2_ and CHA_2_DS_2_-VASc scores with atrial high rate episode

Receiver operating characteristic curves showed that the AUC values of the CHADS_2_ and CHA_2_DS_2_-VASc scores for development of any burden of AHRE were low, ranging from 0.50 to 0.54, although there was a trend suggesting a relationship between the CHA_2_DS_2_-VASc score and AHRE >24 h (AUC 0.54, 95% CI 0.50–0.58, *P *=* *0.053; Supplementary material online, *Table* *S3*). There was no significant difference in AUC values between the CHADS_2_ and CHA_2_DS_2_-VASc scores (*P *=* *0.131 for AHRE ≤6 min, *P *=* *0.679 for AHRE >6 min to ≤6 h, *P *=* *0.137 for AHRE >6 to ≤24 h, and *P *=* *0.768 for AHRE >24 h).

## Discussion

The main finding of this study is that in patients with ICD or CRT-D devices capable of continuous arrhythmia detection, specific patient characteristics are associated with the duration of atrial tachyarrhythmias. Older age and a history of AF or AFL were risk factors for AHRE of longer duration. Furthermore, hypertension showed a strong impact on the development of AHRE lasting >24 h rather than age.

In this population, AHRE between 6 min and 6 h was the most frequently detected duration. In previous studies, reported incidence rates of AHRE ranges from 30% to 70%,^5^ although incidence seems to depend on the clinical characteristics of each study population. An increased risk of adverse outcomes such as ischaemic stroke or systemic embolism has been associated with AHRE >5–6 min.^4^ Whether AHRE have the same implications for antithrombotic prophylaxis as overt AF remains unclear.^14^ Cohort studies have demonstrated that stroke risk in untreated patients with AHRE increases with an increasing burden of AHRE,^15^ and the net benefit of anticoagulation therapy for patients with AHRE >24 h is under investigation in clinical trials.^16^^,^^17^ Anticoagulation may be considered for selected patients with longer burdens of AHRE (e.g. >24 h), based upon anticipated clinical benefit, once patient preferences are also weighed. Clinical practice guidelines recommend careful monitoring of patients with device-detected AHRE, including remote monitoring systems, especially for patients with greater burdens of AHRE and high-risk clinical profiles.^10^

Previous study demonstrated that patients who developed AHRE >24 h showed a significantly higher risk of stroke or systemic embolism compared with those without AHRE, but other durations of AHRE did not show the association with an increased risk.^15^ In the present study, we found significant associations of AHRE >24 h with age, history of AF or AFL, and hypertension. Among those risk factors, age was associated with AHRE >24 h with a HR of 1.59, while hypertension showed more than two-hold risk for AHRE >24 h. This result may suggest that blood pressure may be an important target as one of the modifiable clinical factors for the prevention from adverse outcomes such as stroke or systemic embolism in patients who develop AHRE. Furthermore, stratification of patients according to AHRE duration disclosed a sex-based difference, as women were less likely to exhibit AHRE from 6 min to 24 h duration. Despite a lower age-adjusted prevalence of overt AF in women compared with men, a difference in AHRE based on gender has not been previously reported and warrants confirmation in future studies. The association between use of digoxin and AHRE >24 h is difficult to explain in the absence of data on serum digoxin dosage, plasma concentration, and indications for therapy. Indeed, at supra-therapeutic blood concentrations, digoxin is associated with an increased risk of adverse effects.^18^ We observed a negative association between short AHRE (<6 min) and heart failure, but the clinical relevance of such short AHRE is not well established because AHRE lasting <6 min was previously reported to have low positive predictive value for actual AF episodes.^19^ In addition, the LOOP (Atrial Fibrillation Detected by Continuous ECG Monitoring Using Implantable Loop Recorder to Prevent Stroke in High-risk Individuals) study showed that only a minority (16%) of short episodes of subclinical AF progressed to longer episodes >24 h, and that 22% of patients with AF detected by implantable loop recorder had no other episodes of AF in a 3-year follow-up.^20^ Thus, we cautiously interpret the results regarding the associations between short AHRE and clinical characteristics.

Use of the CHADS_2_ and CHA_2_DS_2_-VASc scores has extended beyond assessment of thromboembolism risk to predict new-onset AF, left atrial remodelling, and AF recurrence after catheter ablation.^21–23^ We found no significant difference in the performance of the two scores to predict AHRE. In fact, unlike clinically manifest AF, these risk scores did not perform well in predicting device-detected AHRE of any duration. One explanation may be that AHRE relate more closely to arrhythmogenic atrial pathology than to thrombogenicity and risk of clinical ischaemic events.

### Limitations

The present study has several limitations. First, the study population consisted of high-risk patients with ICD or CRT-D devices, who often had heart failure or other underlying heart disease associated with a risk of life-threatening arrhythmias. Their clinical characteristics may therefore differ from patients with other types of CIEDs, such as pacemakers and loop recorder. Although we investigated the relationship between clinical characteristics and the duration of AHRE, the association of AHRE with clinical outcomes, including stroke, heart failure, hospitalization, or mortality was not addressed because the IMPACT study randomly assigned patients to anticoagulation based on remote rhythm monitoring compared to conventional follow-up, which may affect the relationship between AHRE and clinical outcomes. Furthermore, we observed a significant difference in the follow-up durations between AHRE <6 min and AHRE >24 h, suggesting that long-term observation may more frequently detect longer AHRE duration during follow-up. Hence, the follow-up duration for each individual could possibly contribute to the results from this study, although there was no significant difference in the follow-up duration between other AHRE duration subgroups. Moreover, in the IMPACT study, AHREs were subjected to an adjudication by the expert committee in the case of events that would have triggered the initiation of anticoagulants. Although some of the AHREs might by chance be due to the false reading by CIEDs, longer AHREs were in general were subjected to an independent adjudication.

## Conclusions

The duration of AHRE varies according to the clinical characteristics of patients with implanted defibrillator or resynchronization devices. Atrial high rate episode >6 min to ≤6 h was most prevalent among all AHRE duration categories in patients with ICD or CRT-D. Longer AHREs more frequently occurred in patients at risk of thromboembolism. Age and history of AF/AFL were risk factors for AHRE >6 min. Furthermore, hypertension showed a strong impact on the development of AHRE >24 h rather than age.

## Supplementary material

Supplementary material is available at *Europace* online.

## Supplementary Material

euab186_Supplementary_Data

## Notes

### Data availability

The data underlying this article were provided by BIOTRONIK under licence. Data will be shared on request to the corresponding author with permission of BIOTRONIK.

## References

[1] Vermond RA , Geelhoed B, Verweij N, Tieleman RG, Van der Harst P, Hillege HL et al Incidence of atrial fibrillation and relationship with cardiovascular events, heart failure, and mortality: a community-based study from the Netherlands. J Am Coll Cardiol 2015;66:1000–7. 26314526 10.1016/j.jacc.2015.06.1314

[2] Gladstone DJ , Spring M, Dorian P, Panzov V, Thorpe KE, Hall J et al Atrial fibrillation in patients with cryptogenic stroke. N Engl J Med 2014;370:2467–77. 24963566 10.1056/NEJMoa1311376

[3] Grau AJ , Weimar C, Buggle F, Heinrich A, Goertler M, Neumaier S et al Risk factors, outcome, and treatment in subtypes of ischemic stroke: the German Stroke Data Bank. Stroke 2001;32:2559–66. 11692017 10.1161/hs1101.098524

[4] Healey JS , Connolly SJ, Gold MR, Israel CW, Van Gelder IC, Capucci A et al Subclinical atrial fibrillation and the risk of stroke. N Engl J Med 2012;366:120–9. 22236222 10.1056/NEJMoa1105575

[5] Freedman B , Boriani G, Glotzer TV, Healey JS, Kirchhof P, Potpara TS. Management of atrial high-rate episodes detected by cardiac implanted electronic devices. Nat Rev Cardiol 17;14:701–14. 28682320 10.1038/nrcardio.2017.94

[6] Miyazawa K , Pastori D, Li Y-G, Székely O, Shahid F, Boriani G et al Atrial high rate episodes in patients with cardiac implantable electronic devices: implications for clinical outcomes. Clin Res Cardiol 2019;108:1034–41. 30759274 10.1007/s00392-019-01432-y PMC6694071

[7] Pastori D , Miyazawa K, Li Y, Székely O, Shahid F, Farcomeni A et al Atrial high-rate episodes and risk of major adverse cardiovascular events in patients with cardiac implantable electronic devices. Clin Res Cardiol 20;109:96–102. 31144064 10.1007/s00392-019-01493-z

[8] Gonzalez M , Keating RJ, Markowitz SM, Liu CF, Thomas G, Ip JE et al Newly detected atrial high rate episodes predict long-term mortality outcomes in patients with permanent pacemakers. Heart Rhythm 2014;11:2214–21. 25131667 10.1016/j.hrthm.2014.08.019

[9] Gorenek B, Bax J, Boriani G, Chen SA, Dagres N, Glotzer TV et al. Device-detected subclinical atrial tachyarrhythmias: definition, implications and management-an European Heart Rhythm Association (EHRA) consensus document, endorsed by Heart Rhythm Society (HRS), Asia Pacific Heart Rhythm Society (APHRS) and Sociedad Latinoamericana de Estimulación Cardíaca y Electrophysiology (SOLEACE). *Europace* 2017;19:1556-78. 10.1093/europace/eux163 28934408

[10] Hindricks G , Potpara T, Dagres N, Arbelo E, Bax JJ, Blomström-Lundqvist C et al; ESC Scientific Document Group. ESC Guidelines for the diagnosis and management of atrial fibrillation developed in collaboration with the European Association of Cardio-Thoracic Surgery (EACTS). Eur Heart J 2021;42:373–498. 32860505 10.1093/eurheartj/ehaa612

[11] Pastori D , Miyazawa K, Li Y, Shahid F, Hado H, Lip GYH. Inflammation and the risk of atrial high-rate episodes (AHREs) in patients with cardiac implantable electronic devices. Clin Res Cardiol 2018;107:772–7. 29667016 10.1007/s00392-018-1244-0 PMC6105258

[12] Martin DT , Bersohn MM, Waldo AL, Wathen MS, Choucair WK, Lip GYH et al Randomized trial of atrial arrhythmia monitoring to guide anticoagulation in patients with implanted defibrillator and cardiac resynchronization devices. Eur Heart J 2015;36:1660–8. 25908774 10.1093/eurheartj/ehv115

[13] DeLong ER , DeLong DM, Clarke-Pearson DL. Comparing the areas under two or more correlated receiver operating characteristic curves: a nonparametric approach. Biometrics 88;44:837–45. 3203132

[14] Bertaglia E , Blank B, Blomström-Lundqvist C, Brandes A, Cabanelas N, Dan G-A et al Atrial high-rate episodes: prevalence, stroke risk, implications for management, and clinical gaps in evidence. Europace 2019;21:1459–67. 31377792 10.1093/europace/euz172 PMC6788209

[15] Van Gelder IC , Healey JS, Crijns HJGM, Wang J, Hohnloser SH, Gold MR et al Duration of device-detected subclinical atrial fibrillation and occurrence of stroke in ASSERT. Eur Heart J 17;38:1339–44. 28329139 10.1093/eurheartj/ehx042

[16] Lopes RD , Alings M, Connolly SJ, Beresh H, Granger CB, Mazuecos JB et al Rationale and design of the apixaban for the reduction of thrombo-embolism in patients with device-detected sub-clinical atrial fibrillation (ARTESiA) trial. Am Heart J 2017;189:137–45. 28625370 10.1016/j.ahj.2017.04.008

[17] Kirchhof P , Blank BF, Calvert M, Camm AJ, Chlouverakis G, Diener H-C et al Probing oral anticoagulation in patients with atrial high rate episodes: rationale and design of the Non-vitamin K antagonist Oral anticoagulants in patients with Atrial High rate episodes (NOAH-AFNET 6) trial. Am Heart J 2017;190:12–8. 28760205 10.1016/j.ahj.2017.04.015 PMC5546174

[18] Pastori D , Carnevale R, Nocella C et al Digoxin and platelet activation in patients with atrial fibrillation: in vivo and in vitro study. J Am Heart Assoc 2018;7:e009509. 30571484 10.1161/JAHA.118.009509 PMC6404445

[19] Kaufman ES , Israel CW, Nair GM, Armaganijan L, Divakaramenon S, Mairesse GH et al; ASSERT Steering Committee and Investigators. Positive predictive value of device-detected atrial high-rate episodes at different rates and durations: an analysis from ASSERT. Heart Rhythm 12;9:1241–6. 22440154 10.1016/j.hrthm.2012.03.017

[20] Diederichsen SZ , Haugan KJ, Brandes A, Lanng MB, Graff C, Krieger D et al Natural history of subclinical atrial fibrillation detected by implanted loop recorders. J Am Coll Cardiol 2019;74:2771–81. 31779791 10.1016/j.jacc.2019.09.050

[21] Chao T-F , Lin Y-J, Tsao H-M, Tsai C-F, Lin W-S, Chang S-L et al CHADS(2) and CHA(2)DS(2)-VASc scores in the prediction of clinical outcomes in patients with atrial fibrillation after catheter ablation. J Am Coll Cardiol 2011;58:2380–5. 22115643 10.1016/j.jacc.2011.08.045

[22] Li Y , Ding W, Wang H, Song N, Lin L, Wang Z et al Relationship of CHA2DS2-VASc and CHADS2 score to left atrial remodeling detected by velocity vector imaging in patients with atrial fibrillation. PLoS One 2013;8:e77653. 24147047 10.1371/journal.pone.0077653 PMC3798687

[23] Saliba W , Gronich N, Barnett-Griness O, Rennert G. Usefulness of CHADS2 and CHA2DS2-VASc scores in the prediction of new-onset atrial fibrillation: a population-based study. Am J Med 2016;129:843–9. 27012854 10.1016/j.amjmed.2016.02.029

