# Supplementary material for: Characteristics of patients with atrial high rate episodes detected by implanted defibrillator and resynchronization devices
Source: Europace. 2021 Aug 24;24(3):375–83. doi: 10.1093/europace/euab186 (PMC8892042; doi:10.1093/europace/euab186)
Supplement: euab186_Supplementary_Data [file euab186_supplementary_data.docx]

Supplementary Table 1. Time frame over AHRE documentation

|  | | AHRE groups | | | |
| --- | --- | --- | --- | --- | --- |
|  | | ≤ 6 min  (n=292) | >6 min/≤6 hours  (n=284) | >6 hours/≤24 hours  (n=187) | >24 hours  (n=182) |
| AHRE ≤6 min | Days of AHRE detection, days | 4.3 ± 13.5 | 12.7 ± 32.0 | 45.1 ± 101.0 | 150.3 ± 223.7 |
|  | Fraction of AHRE detection during F/U, % | 0.6 ± 1.5 | 2.2 ± 6.2 | 5.9 ± 11.2 | 16.6 ± 20.6 |
| AHRE >6 min | Days of AHRE detection, days | NA | 4.7 ± 8.5 | 29.3 ± 70.6 | 143.0 ± 220.7 |
|  | Fraction of AHRE detection during F/U, % | NA | 0.8 ± 1.9 | 4.1 ± 8.8 | 15.8 ± 20.5 |
| AHRE >6 hr | Days of AHRE detection, days | NA | NA | 7.7 ± 14.4 | 128.4 ± 215.8 |
|  | Fraction of AHRE detection during F/U, % | NA | NA | 1.6 ± 5.8 | 14.4 ± 20.2 |
| AHRE >24 hr | Days of AHRE detection, days | NA | NA | NA | 2.08 ± 2.3 |
|  | Fraction of AHRE detection during F/U, % | NA | NA | NA | 0.3 ± 0.2 |

Supplementary Table 2. Clinical factors associated with the development of each burden of AHRE in patients without history of AF/AFL

|  | ≤ 6 minutes  (n=292) | | >6 min/≤6 hours  (n=284) | | >6 hours/≤24 hours  (n=187) | | >24 hours  (n=182) | |
| --- | --- | --- | --- | --- | --- | --- | --- | --- |
|  | HR (95% CI) | P value | HR (95% CI) | P value | HR (95% CI) | P value | HR (95% CI) | P value |
| Age ≥ 65 years | 0.85 (0.65–1.11) | 0.238 | 1.76 (1.34–2.31) | <0.001 | 1.52 (1.08–2.14) | 0.017 | 1.44 (0.99–2.09) | 0.054 |
| Female gender | 1.04 (0.78–1.39) | 0.803 | 0.68 (0.49–0.94) | 0.020 | 0.63 (0.41–0.96) | 0.034 | 0.73 (0.47–1.13) | 0.156 |
| Hypertension | 1.06 (0.73–1.54) | 0.756 | 0.76 (0.54–1.05) | 0.094 | 1.02 (0.64–1.62) | 0.946 | 1.87 (0.97–3.61) | 0.063 |
| Diabetes mellitus | 1.01 (0.77–1.33) | 0.941 | 0.97 (0.74–1.28) | 0.826 | 0.93 (0.66–1.33) | 0.701 | 1.31 (0.91–1.90) | 0.148 |
| Heart failure | 0.67 (0.44–1.01) | 0.058 | 0.95 (0.60–1.51) | 0.816 | 0.83 (0.47–1.47) | 0.521 | 1.14 (0.57–2.29) | 0.716 |
| History of stroke/TIA | 1.24 (0.81–1.92) | 0.326 | 0.85 (0.53–1.37) | 0.501 | 0.84 (0.46–1.54) | 0.579 | 0.84 (0.45–1.57) | 0.583 |
| Vascular disease | 0.92 (0.68–1.26) | 0.618 | 0.88 (0.65–1.19) | 0.395 | 0.99 (0.67–1.48) | 0.968 | 0.75 (0.50–1.14) | 0.180 |
| Non-ischemic CM | 1.17 (0.88–1.57) | 0.284 | 1.03 (0.77–1.37) | 0.866 | 1.04 (0.71–1.52) | 0.848 | 0.71 (0.47–1.09) | 0.118 |
| Valvular disease | 1.19 (0.91–1.55) | 0.202 | 1.10 (0.85–1.43) | 0.462 | 0.89 (0.63–1.24) | 0.483 | 1.09 (0.76–1.57) | 0.648 |
| Beta-blockers | 0.82 (0.51–1.31) | 0.405 | 0.79 (0.50–1.25) | 0.315 | 0.92 (0.49–1.73) | 0.802 | 0.72 (0.37–1.37) | 0.314 |
| ACE-I/ARB | 1.28 (0.86–1.90) | 0.221 | 1.30 (0.88–1.91) | 0.186 | 1.36 (0.80–2.29) | 0.255 | 1.45 (0.80–2.62) | 0.224 |
| Digoxin | 1.07 (0.68–1.57) | 0.717 | 1.35 (0.95–1.92) | 0.095 | 1.17 (0.72–1.90) | 0.524 | 1.30 (0.77–2.16) | 0.306 |
| AAD | 0.77 (0.45–1.32) | 0.346 | 0.76 (0.45–1.28) | 0.293 | 0.93 (0.49–1.76) | 0.820 | 0.78 (0.37–1.63) | 0.506 |
| Statin | 0.85 (0.62–1.17) | 0.317 | 0.94 (0.68–1.30) | 0.719 | 1.13 (0.73–1.75) | 0.585 | 1.31 (0.81–2.12) | 0.272 |

CI; confidence intervals, HR; hazard ratio

Supplementary Table 3. Predictive ability of CHADS_2_ and CHA_2_DS_2_-VASc score for developing of AHRE

|  | AUC (95% CI) | P value for AUC | P value for AUC comparison |
| --- | --- | --- | --- |
| AHRE ≤ 6 min | | | |
| CHADS_2_ | 0.52 (0.48–0.55) | 0.477 | 0.131 |
| CHA_2_DS_2_-VASc | 0.53 (0.50-0.57) | 0.103 |  |
| 6 min < AHRE ≤ 6 hr | | | |
| CHADS_2_ | 0.52 (0.48-0.55) | 0.544 | 0.679 |
| CHA_2_DS_2_-VASc | 0.50 (0.46-0.54) | 0.965 |  |
| 6 hr < AHRE ≤ 24 hr | | | |
| CHADS_2_ | 0.50 (0.46-0.54) | 0.934 | 0.137 |
| CHA_2_DS_2_-VASc | 0.52 (0.47-0.56) | 0.552 |  |
| AHRE > 24 hr | | | |
| CHADS_2_ | 0.54 (0.50–0.58) | 0.152 | 0.768 |
| CHA_2_DS_2_-VASc | 0.54 (0.50–0.58) | 0.053 |  |

AUC; area under curve

*CHADS_2_ score as a reference
